# Commencements

**Published:** 1892-08

**Authors:** 


### Commencements.

### German—American Dental College.

The annual commencement exercises of the German American Dental College of Chicago, Ills., were held in the college building, on March 26th, 1892.

The number of matriculates for the session was ten.

The degree of Doctor of Dental Surgery was conferred on H. Schnitker.

### University of Pennsylvania—Department of Dentistry.

The thirteenth annual commencement of the Dental Department of the University of Pennsylvania was held at the American Academy of Music, of Philadelphia, Pa., on May 6, 1892.

The number of matriculates for the session was one hundred and sixty-nine.

An interesting address was delivered by John Guiteras, M.D., Professor of General Pathology and Morbid Anatomy.

The degree of D.D.S. was conferred by William Pepper, M. D., LL.D., Provost of the University, upon the following:

| NAME. | RESIDENCE. |
|---|---|
| Armada, Carlos A. de, | Brazil |
| Ayers, Josiah | P. Ed. Island |
| Baer, Harry K. | Pennsylvania |
| Beitzel, Walter G. | Kansas |
| Benninghoff, Joseph L. | Pennsylvania |
| Berger, Johannes | Germany |
| Bodine, Fred. M | Pennsylvania |
| Bond, Francis H. | Pennsylvania |
| Bonwill, Edward W | Pennsylvania |
| Bowen, John J | Rhode Island |
| Brown, Andrew Law | Connecticut |
| Christensen, Wilhelm E | Denmark |
| Clark, Frank T | Pennsylvania |
| Coen, Edward C | Illinois |
| Condict, Frederick L | New Jersey |
| Cook, Frank P. | Pennsylvania |
| Cooper, William M | Pennsylvania |
| Crankshaw, Charles W. | Pennsylvania |
| Danforth, Joseph T | Pennsylvania |
| Diefenderfer, Victor H. | Pennsylvania |
| Dreher, Jeremiah H. | North Carolina |
| Eldredge, J. Smallwood | New Jersey |
| Ernsmere, John B | New York |
| Flexer, Richard J | Pennsylvania |
| Gearhart, J. Beaver | Pennsylvania |
| Gilbert, Lewis H. | New York |
| Goddard, Henry Ernest | England |
| Grubb, A. Herbert | Pennsylvania |
| Gunn, William | New Zealand |
| Hamilton, Harry B | New York |
| Hart, Arch Combs | California |
| Hause, Edward B | Pennsylvania |
| Haynes, Melvin G | New York |
| Hine, Robert H | Connecticut |
| Holmes, Walter T | Connecticut |
| Hotz, Paul | Switzerland |
| Ives, A. Scott | Canada |
| Joachim, Edward B | Pennsylvania |
| Johnson, George H. jr. | Bahamas |
| Johnson, Oakley | Washington |
| Johnston, H. Frank | Canada |
| Kittams, James H | New York |
| Kniewel, Johannes | Germany |
| Landsberg, Bernhard | Germany |
| Lapp, Elbert W. | Pennsylvania |
| Lawton, Burtis E | Nebraska |
| Lemker, W. J. ter Kuile | Holland |
| Leonhardi, Charles J. | California |
| Le Suer, William J | New York |
| Ligonde, Louis G | Hayti |
| Lopez, Jose Lucio | Central America |
| Lukens, Clarence D. | Iowa |
| Lynch, Patrick F | Pennsylvania |
| Matthey, Edouard | Switzerland |
| Middaugh, W. Clay | Pennsylvania |
| Mitchell, Vethake E. | Ohio |
| Murto, Frank D | Pennsylvania |
| Nellis, George H | New York |
| Newgarden, Charles | Pennsylvania |
| Parshall, Edward E | Pennsylvania |
| Phipps, Harry D | Texas |
| Protsman, Albert B | Indiana |
| Richards, John W | Pennsylvania |
| Ridgway, Shessie Worth | Pennsylvania |
| Ross, Alfred T | Pennsylvania |
| Rymer, James Francis | England |
| Saunders, T. Darwin | New York |
| Schoff, P. Frank | Pennsylvania |
| Segar, J. Clark | Connecticut |
| Seymour, Robert J | Canada |
| Skillman, E. Harvey | New York |
| Smith, A. Fowler | New York |
| Sowash, W. Harry | Pennsylvania |
| Stathers, James R | West Virginia |
| Stewart, Charles A | Pennsylvania |
| Swing, Harry R | Pennsylvania |
| Wardell, Frank C | Pennsylvania |
| Whitlock, Edward P | Pennsylvania |
| Whitmer, S. Edwin | Pennsylvania |
| Willis, Albert Lincoln | Washington |
| Wimmer, George J | Pennsylvania |
| Windmuller, Percival | Germany |
| Witthaus, Carl. | Germany |
| Wuensche, Eugen C | Germany |

JUNE 11, 1891.

| | |
|---|---|
| Adams, Philip W | Massachusetts |
| Foster, A. Lee | Pennsylvania |
| Horter, William B. | Pennsylvania |
| Lawton, George A | Connecticut |
| McCarthy, Thomas A | New Hampshire |
| Macdonald, Robert | Australia |
| O'Bourke, John M | Cuba |
| Ulrich, George R | Pennsylvania |

Total 92.

### Boston Dental College.

The twenty-fifth annual commencement exercises of the Boston Dental College were held in Berkeley Temple, Wednesday, June 15, 1892, at 7:30 P. M. An address was delivered by B. S. Ladd, Esq., and the valedictory by Ellsworth N. Brown, D.D.S.

The degree of D.D.S. was conferred on the following graduates by the President of the College, I. J. Wetherbee, D.D.S.:

| NAME. | NAME. |
|---|---|
| John Charles Fremont Bridge | George Nelson Johnson |
| Karl Schurz Brock | Philip Patrick Kelley |
| Ellsworth Newton Brown | Albert Henry Ketcham |
| Richard Bullock Callaway | John Stephen King |
| Thomas Patrick Cahill | Irving Miles Luce |
| Stephen Harry Chase | John Fletcher Maloney |
| Pearl Raymond Copeland | William Rodney Marsh |
| Thomas Francis Cuff | Frank Rittle Mayers |
| William Vaughan Davies | Norry Miett |
| Albert Jones Derby | Louis Dearborn Millett |
| William Henry Duddy Boston | William Samuel Pearman |
| Walter Lovett Dunton | Charles Nahum Piper |
| Arthur Ellis Estebrooke | Edwin Alexander Quinn |
| James Andrew Ewing | Frederick Alexander Robinson |
| John Wood Forbes | Julius Stahl |
| *Guy Norman Gammon | William Pray Swasey |
| Daniel Griffin | George Lawrence True |
| William Irving Hervey | John Edward Walsh |
| *Mozes Jessurun | Clarance Parker Whittle |

Total 38.

* Guy Norman Gammon and Mozes Jessurun have fulfilled all the require ments, but not being twenty-one years of age cannot now receive the degree.

### Harvard University—Dental Department.

The annual commencement exercises of the Dental Department of the Harvard University were held in connection with those of the other departments of that institution on June 29th, 1892, in Sander's Hall, Cambridge, Mass.

The number of matriculates for the session was forty-nine.

The degree of D.D.S. was conferred upon the following graduates by the President of the University, Chas. W. Elliott, L.L.D.:

| NAME. | NAME. |
|---|---|
| Edward Stanley Bryant | Herbert Frederick Hill |
| Allen Stanley Burnham | Albert Edward Hulme |
| Carol Edward Bugbee Chase | Richard Carol Moritz |
| Willard Eben Curtice | Henry Snow Parsons |
| Kirk Addison Davenport, D.D.S | Henry Robinson Peach |
| Ernest Frederick Gabell | Henry Edward Rose |
| Theodore Hallett | Nathan Prindle Wyllie |

Total 14.

### College of Dental Surgery of the University of Michigan.

The seventeenth annual commencement of this department was held in University Hall, Ann Arbor, Thursday, June 30th, 1892.

The number of matriculates for the session was one hundred and eighty-four.

The annual commencement oration was delivered by Justin Winsor, L.L.D., of Harvard University.

The degree of D.D.S. was conferred on the following candidates by the President of the University, James B. Angell, L.L.D.:

NAME.

Burt Abell
Samuel Howard Arthur
Harry Howard Avery
Harry Park Ball
Walter Joel Bell
Charles Lee Blunt
Herbert Warren Bovee
Charles Edward Burchfield
Charles Sylvester Chadwick
Timothy Spencer Childs
Thomas Coleman, D.D.S.,
Royal College of Dental Surgeons
Eli Mahlon Conrad
Oscar Willmott Daly, D.D.S.,
Royal College of Dental Surgeons
Archibald Warren Diack
George Dilworth
Elmer C. Goldthorp
Allison William Haidle
Charles William Hall
Henry James Harvey
May Weston

NAME.

Thomas Ebenezer Howson
Osgood Frank Ingalls
Vida Annette Latham
Ben. Hubbard Lee
Frank P. Martin
James Andrew Milliken, D.D.S.,
University of Pennsylvania
Henry Milling
John Albert Moore
William James Mummery
William Edward Prather, D.D.S.,
University of Maryland
Frank S. Prettyman
Ellen Dennison Searle
Edward Douglass Slawson
Joseph Allen Snyder
Edward Bartlett Spalding
Carrie Marsden Stewart
George Ernest Tribby
Anthony Van Kammen
Austin Smith Watrous
Total 39.

### Howard University—Dental Department.

The annual commencement exercises of The Dental Department of Howard University, were held at the Congregational Church, Washington, D. C., on Wednesday evening, April 13, 1892.

The annual address was delivered by Prof. D. S. Lamb.

The number of matriculates for the session was ten.

The degree of D.D.S. was conferred on the following graduates by Rev. J. Eames Rankin, D.D., LL.D., President of the Board of Trustees:

| NAME. | RESIDENCE. |
|---|---|
| Andrew Gwathney | Virginia |
| Albert S. Johnson | New York |
| John McDonald | P. Ed. Island |

Total 3.

### University of Minnesota—Dental Department.

The annual commencement exercises of the Dental Department were held in connection with those of the other departments of the University of Minnesota, on Thursday, June 2nd, 1892.

The degree of Doctor of Dental Surgery was conferred upon the following by Cyrus Northrop, L. L. D., President of the University: Frank W. Force, Miland A. Knapp, James W. Paul, Thomas F. Williams.

